# Mathematical Optimization of the Combination of Radiation and Differentiation Therapies for Cancer

**DOI:** 10.3389/fonc.2013.00052

**Published:** 2013-03-18

**Authors:** Jeff W. N. Bachman, Thomas Hillen

**Affiliations:** ^1^Department of Mathematical and Statistical Sciences, Centre for Mathematical Biology, University of AlbertaEdmonton, AB, Canada

**Keywords:** cancer stem cells, tumor stem cells, radiation treatment, differentiation therapy, combination therapy, cancer modeling

## Abstract

Cancer stem cells (CSC) are considered to be a major driver of cancer progression and successful therapies must control CSCs. However, CSC are often less sensitive to treatment and they might survive radiation and/or chemotherapies. In this paper we combine radiation treatment with differentiation therapy. During differentiation therapy, a differentiation promoting agent is supplied (e.g., TGF-beta) such that CSCs differentiate and become more radiosensitive. Then radiation can be used to control them. We consider three types of cancer: head and neck cancer, brain cancers (primary tumors and metastatic brain cancers), and breast cancer; and we use mathematical modeling to show that combination therapy of the above type can have a large beneficial effect for the patient; increasing treatment success and reducing side effects.

## 1 Introduction

Cancer stem cells (CSC) have been identified in many cancer types as the driving force behind cancer growth and progression (Dick, 2003; Singh et al., 2003; Sell, 2004; Todaro et al., 2007; Maitland and Colling, 2008. Dingli and Michor (2006) attested as title of their 2006 paper that “*Successful therapy must eradicate cancer stem cells*.” This is hard to do, since CSC can be found at any location in the tumor (Youssefpour et al., 2012) and they are difficult to identify *in vivo* (Kummermehr, 2001). Furthermore, cancer stem cells are less sensitive to radiation or other cell killing agents (Kim and Tannock, 2005; Pajonk et al., 2010). One method to sensitize cancer stem cells is to use differentiation promoting growth factors that force CSCs to differentiate and become more sensitive to radiation. Possible differentiation promoters, which are discussed in the literature, are members of the TGF-*β* superfamily (Transforming growth factor – *β*; see Lander et al., 2009; Watabe and Miyazono, 2009; Meulmeester and Ten Dijke, 2011). TGF-*β* is known to increase stem cell differentiation, but it also affects other characteristics of growing tumors such as invasion and immune evasion. Here we focus on the differentiation stimulating properties of TGF-*β*. Other examples of differentiation therapy agents include ATRA-therapy (all-trans-retinoic acid) for acute promyelocytic leukemia (Sell, 2004) and a combination of INF-*β* (inferon-beta) and MEZ (mezerein) for treatment of melanoma (Leszczyniecka et al., 2001). Many more agents are currently investigated for their differentiation promoting activities (Leszczyniecka et al., 2001).

The mathematical modeling of cancer progression and treatment has a long history and individual treatments as well as combination therapies have been studied. A comprehensive review is given in Swierniak et al. (2009).

Our modeling and analysis of *differentiation therapy* and the combination with *radiation* *therapy* was motivated through a detailed computational model of Youssefpour et al. (2012). The model of Youssefpour et al. (2012) consist of a coupled system of partial differential equations for CSC, transient amplifying cells (TAC), differentiated cancer cells (DC), growth factors and growth inhibiting factors, and differentiation promoters. In addition, the model is spatially explicit and physical properties related to pressure and force balances are included. This model was developed over a series of publications (see Wise et al., 2008 and references therein).

Youssefpour et al. (2012) combine the detailed cancer model with differentiation therapy and with radiation therapy. They find that an appropriate combination of differentiation therapy and radiation therapy can control the cancer in situations where each individual treatment would fail. Their treatment terms are generic terms for differentiation and radiation treatments and they have not been modeled for a specific cancer type. The goal of this paper is to challenge Youssefpour’s findings for the specific cases of *head and neck cancer*, *brain cancers*, and *breast cancer*. We adapt the model of Youssefpour et al. (2012) to be able to include realistic growth and death rates, realistic differentiation therapies, realistic radiation therapy schedules, and appropriate tissue dependent radio-sensitivities. We sacrifice, however, the spatial structure of the model and we study the well mixed, spatially homogeneous situation. We argue that if the effect of combination therapy can be clearly demonstrated on a simpler model, then this mechanism will be part of a more complicated model as well. We find that for average parameters of brain cancers and for breast cancer we can confirm the finding of Youssefpour et al. (2012) in that, combination therapy can control a tumor, where each individual method would fail. For head and neck cancer, we find that differentiation therapy can drastically reduce the amount of radiation that is needed to control the tumor.

## 2 Materials and Methods

We use mathematical modeling and numerical simulations to predict the outcome of these therapies. Our mathematical model is based on a model for cancer stem cells that was derived in Hillen et al. (2013). It describes the interplay of cancer stem cells *U*(*t*) and non-stem cancer cells *V*(*t*). To describe radiation therapy we use the well-established linear quadratic model (see Fowler, 1989) with realistic standard treatments (five treatments per week, weekends off) and with tissue specific radiosensitivity parameters *α* and *β* (see Fowler, 1989). The parameterization of differentiation therapy is more difficult, since differentiation promoters are hard to quantify. Here we use the model and parameters of Youssefpour et al. (2012).

### 2.1 The mathematical model

We begin with the spatially homogeneous, cancer stem cell model developed by Hillen et al. (2013). By spatial homogeneity, we mean that cell density, cell growth, and the distribution of chemicals are homogeneous throughout the tumor region.

(1) $$\begin{array}{rcl} \overset{\cdot }{U}\left(t\right) & =\delta m_{U}k\left(P\left(t\right)\right)U\left(t\right) & \end{array}$$

(2) $$\begin{array}{rcll} \overset{\cdot }{V}\left(t\right) & =\left(1-\delta \right)m_{U}k\left(P\left(t\right)\right)U\left(t\right) & & \\ & \;+m_{V}k\left(P\left(t\right)\right)V\left(t\right)-a_{V}V\left(t\right) & \end{array}$$

where *U*(*t*) is the volume fraction of cancer stem cells (CSCs) with respect to the total domain of interest, which contains both tumor and host cells. Similarly, *V*(*t*) is the volume fraction of non-stem tumor cells (TCs) with respect to the total domain of interest. The total volume fraction of the tumor is represented by *P*(*t*), that is, *P*(*t*) = *U*(*t*) + *V*(*t*). The parameter *δ* is the probability that a CSC will give rise to another CSC, when it divides. Thus, 1 − *δ* is the probability that a CSC will give rise to one CSC and one TC, when it divides. It is assumed that the parent CSC remains (Sell, 2004). The growth rates of the CSCs and TCs are given by *m_U_* and *m_V_*, respectively. The apoptosis rate of the TCs is represented by *a_V_*; we assume that CSCs do not undergo apoptosis since they have unlimited replicative potential. Cell growth and differentiation are tempered by *k*(*P*(*t*)), which is essentially a volume constraint. Hillen et al. (2013) assume that *k*(*P*) is monotonically decreasing in *P* and piecewise differentiable, and they set *k*(*P*) > 0 for *P* ∈ [0,P*) and *k*(*P*) = 0 for all *P* ≥ P*, for some P* > 0. For the purposes of this paper, we adopt the version of *k*(*P*) used by Hillen et al. (2013) and assume normalization of P* = 1 limiting *P* to a maximum volume fraction of one, and *k*(0) = 1. For simulations we use:

(3) $$k\left(P\right)=\max\left\{1-P^{4},0\right\}$$

To match the notation used by Youssefpour et al. (2012) we set *δ* = 2*p* − 1. It follows that 1 − *δ* = 2(1 − *p*). In this case, *p* is the probability that a CSC gives rise to two CSCs, rather than two TCs, when it divides. That is, *p* is the probability that a CSC renews itself, and 1 − *p* is the probability that a CSC differentiates. While this model of CSC division ignores asymmetric division, it is equivalent to the model in equations (1) and (2), as shown in the Appendix of Hillen et al. (2013). The resulting model is given in equations (4) and (5).

(4) $$\begin{array}{rcl} \overset{\cdot }{U}\left(t\right) & =\;\left(2p-1\right)m_{U}k\left(P\left(t\right)\right)U\left(t\right) & \end{array}$$

(5) $$\begin{array}{rcll} \overset{\cdot }{V}\left(t\right) & =2\left(1-p\right)m_{U}k\left(P\left(t\right)\right)U\left(t\right) & & \\ & \;+m_{V}k\left(P\left(t\right)\right)V\left(t\right)-a_{V}V\left(t\right) & \end{array}$$

### 2.2 Behavior of the untreated tumor model

As noted in Hillen et al. (2013), if there are no CSCs, the TC population is governed by the equation

$$\overset{\cdot }{V}\left(t\right)=m_{V}k\left(V\left(t\right)\right)V\left(t\right)-a_{V}V\left(t\right)$$

and since *k* is assumed to be decreasing, the TC population is fated to die out if *m_V_k*(0) < *a_V_*. Further, if we assume that *k* is strictly decreasing, then the TC population dies out if

(6) $$m_{V}k\left(0\right)\leq a_{V}$$

since either *V* = 0 and the TCs are already extinct, or *V* > 0 and so *k*(*V)* < *k*(0) and *m_V_k*(*V)* < *a_V_* for all *V* > 0.

The steady states of the model defined in (1, 2) are discussed in detail in Hillen et al. (2013), where it is assumed that the growth rates *m_U_* and *m_V_* are both one and that the TC apoptosis rate *a_V_* is greater than zero. Here, we give the main results, which also apply to the model as stated in (1, 2) or equivalently in (4, 5). We note that in the untreated tumor, we assume *δ* ∈ (0, 1), that is *p* ∈ (0.5, 1), such that (2*p* − 1) > 0. The steady states of the system are

$$\begin{array}{rcll} X_{0} & =\left(0,0\right),\;X_{V}=\left(0,V_{0}\right),\;X_{U}=\left(1,0\right), & & \\ & \text{with}\;k\left(V_{0}\right)=\frac{a_{V}}{m_{v}}. & & \end{array}$$

The origin, *X*_0_, has eigenvalues *λ*_1_ = (2*p* − 1)*m_U_k*(0) > 0 and *λ*_2_ = *m_V_k*(0) − *a_V_*. Thus, *X*_0_ is an unstable steady state. The TC only steady state, *X_V_*, occurs where *V*_0_ solves *m_V_k*(*V*_0_) = *a_V_* and has eigenvalues *λ*_1_ = (2*p* − 1)*m_U_k*(*V*_0_) > 0 and *λ*_2_ = *m_V_k*′(*V*_0_)*V*_0_. Thus, *X_V_* is also unstable. The linearization for the pure CSC steady state, *X_U_*, has negative trace, *m_U_k*′(1) − *a_V_*, and positive determinant, −*a_V_*(2*p* − 1)*m_U_k*′(1), thus both eigenvalues are negative, and *X_U_* is a stable steady state. Hillen et al. (2013) have shown that *X_U_* is globally asymptotically stable in the biologically relevant region where *U* ∈ [0, 1], *V* ≥ 0, and *U* + *V* ≤ 1.

**Figure 1:**
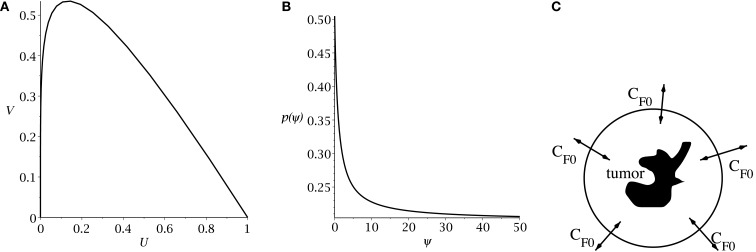
**(A)** Slow manifold from equation (10) in the (*U*, *V*) phase plane. **(B)** rate of symmetric division *p*(*t*) [equation (7)] as a function of the sensitivity *ψ*. The values of *p_max_* and *p_min_* are set to 0.505 and 0.2. The differentiation promoter, *C_F_*, is fixed at one. **(C)** Schematic of the radial diffusion problem for the differentiation promoter *C_F_*.

Hillen et al. (2013) derive the slow manifold of the system defined by (1, 2), where they take *m_U_* = *m_V_* = 1. The slow manifold is a subset of the phase space ((*U*, *V)*-plane) which describes the long time dynamics of the system. As shown in Hillen et al. (2013), solutions very quickly converge to the slow manifold, and then they slowly follow this manifold getting closer to the attractor at (1, 0). This same slow manifold applies to the system defined by equations (4) and (5). We simply restore *m_U_* and *m_V_* and set *δ* = 2*p* − 1, as described above, giving the slow manifold (see Figure 1A):

$$M:=\left\{\left(U,V\right):\left(a_{V}-m_{V}k\left(P\right)\right)V=m_{U}k\left(P\right)U,P=U+V\right\}$$

The main result of Hillen et al. (2013) is the existence of the *Tumor Growth Paradox*. They show that a tumor with larger death rate *a_V_* grows quicker on the slow manifold. As a consequence, tumors with larger death rate outgrow tumors with lower death rate. The reason is that increased TC death can liberate CSC which were surrounded by TC, and it can allow CSC to replicate and produce more CSCs. As a result, the tumor becomes bigger. See Hillen et al. (2013) for the detailed argumentation using geometric singular perturbation analysis of the system.

### 2.3 Modeling of differentiation therapy

Following Youssefpour et al. (2012), we model differentiation therapy through a simple relationship between the average level of the differentiation promoter, which we denote *C_F_*, and the probability of CSC self-renewal, *p*. Unlike the model of Youssefpour et al. (2012) our model does not include a self-renewal promoter; thus, we use the relationship set forth by Youssefpour et al. (2012) but we omit the self-renewal promoting factor:

(7) $$p\left(t\right)=p_{min}+\left(p_{max}-p_{min}\right)\left(\frac{1}{1+\psi C_{F}\left(t\right)}\right)$$

where *p_max_* is the maximum probability of self-renewal, and *p_min_* is the minimum probability of self-renewal. The value of *p_max_* is attained if no differentiation promoter *C_F_* is present, while *p_min_* is attained for *C_F_* → ∞. Youssefpour et al. (2012) choose *p_max_* = 1 and *p_min_* = 0.2 in their therapy simulations. Unlike Youssefpour and coworkers, we do not model the production of differentiation promoters by tumor cells. Thus, *C_F_* solely represents the level of differentiation promoter prescribed during differentiation therapy. To address this lack of endogenous differentiation promoters, we choose *p_max_* = 0.505, which is equivalent to setting *δ* = 0.01, as was done by Hillen et al. (2013). Following Youssefpour et al. (2012) we choose *p_min_* = 0.2. The parameter *ψ* models the sensitivity of the CSCs to the differentiation promoter. The dependence of *p*(*t*) on the sensitivity *ψ* is shown in Figure 1B. Other possible effects of differentiation therapy, such as effects on growth rates, are ignored, as they are by Youssefpour et al. (2012).

To model the average level of differentiation promoter within the spatially homogeneous tumor as a function of time, *C_F_*(*t*), we assume that the tumor resides in a spherical region of tissue and that the differentiation promoter enters this area through the boundary. The ODE system [equations (4) and (5)] gives the mean tumor behavior in this spherical tissue region. Growth promoter that enters the region from the boundary will diffuse very quickly and attain a steady state distribution over this region. To compute this value of *C_F_*(*t*) we solve the problem of diffusion over a sphere of radius *R* and average the solution over the volume of the sphere. A schematic is given in Figure 1C. We use a lower case letter to describe the radial symmetric solution *c_F_*(*r*, *t*) of the following boundary value problem

$$\begin{array}{rcll} \frac{\partial c_{F}}{\partial t} & =\omega \left(\frac{\partial }{\partial r}\left(\frac{\partial c_{F}}{\partial r}\right)+\frac{2}{r}\frac{\partial c_{F}}{\partial r}\right) & & \\ c_{F}\left(R,t\right) & =C_{F0}\left(t\right). & & \end{array}$$

Here *ω* is the effective diffusivity of the differentiation promoter. We set *ω* = 10^−7^ cm^2^/s throughout our simulations. Before differentiation therapy begins, *C*_*F*0_(*t*) is zero. When differentiation therapy begins, the boundary condition on the sphere is set to *C*_*F*0_(*t*) = 1 and the promoter diffuses into the sphere. When differentiation therapy ends, the boundary condition is simply set to zero and the promoter diffuses out of the sphere. We then set

$$C_{F}\left(t\right)=\frac{3}{R^{3}}\int_{0}^{R}c_{F}\left(r,t\right)r^{2}dr.$$

### 2.4 Modeling of radiation therapy

To model external beam fractionated radiotherapy, we apply the broadly used linear quadratic (LQ) model. The surviving fraction of cells, *S*(*d*), after a single fraction of *d* grays (Gy) of radiation, is given by

(8) $$S\left(d\right)=\exp\left(-\alpha d-\beta d^{2}\right)$$

where *α* may be interpreted as lethal damage due to a single track of radiation, and *β* may be interpreted as lethal damage due to the misrepair of DNA damage produced by two separate tracks of radiation (Sachs et al., 2001). As a simple approximation of the radiation resistance of CSCs, we assume that they are better able to repair DNA double strand breaks such that the quadratic interaction term *β* is zero for CSC. We further assume that there is no interaction between DNA damage produced by separate fractions of radiation, owing to the relatively large time between fractions, typically 1 day, when compared to typical DNA repair times on the order of 1 h (O’Rourke et al., 2009).

Rather than incorporate an appropriate form of the LQ model into the system of ODEs [equations (4) and (5)], we simply apply equation (8) to the CSC volume fraction, *U*, and to the TC volume fraction, *V*, at scheduled times during the simulation, using *α* and *β* values appropriate for each cell type. For example, if a fraction is scheduled to be delivered at the beginning of the two-hundredth day of the simulation, the simulation is stopped at this time, the LQ model is applied to *U* and *V*, using their respective parameter values, and the simulation is continued at 200 days plus the fraction duration, using the surviving fractions given by equation (8) as the new initial conditions. We assume fraction durations of 10 min throughout our simulations.

### 2.5 Tumor control probability

We use tumor control probability (TCP) to model the probability that the cells remaining after treatment will die out. To reflect the fact that we must eliminate all CSCs for treatment success (Dingli and Michor, 2006), and the fact that TCs are doomed to die out in the absence of CSCs, we calculate TCP based on the number of CSCs remaining after treatment, using the Poisson TCP formula (see Gong et al., 2013 for Poisson TCP and other TCP models).

(9) $$TCP=\exp\left(-N_{U}\right)\approx \exp\left(-U\frac{4}{3}\pi R^{3}\rho \right)$$

where *N_U_* stands for the number of CSCs; *R* is the radius of the spherical region of interest in cm, as described in the section on differentiation therapy; and *ρ* is the density of cells in the region of interest, which we assume to be 10^9^ cells per cm^3^, a typical cell density for tumors (for example, see Joiner et al., 2009) The closer TCP is to one, the greater the probability that all CSCs die out and the tumor is controlled.

### 2.6 Numerical simulations

For all numerical simulations of the tumor model [equations (4) and (5)] we assume the mitosis rates of the CSCs and TCs are equal. That is, *m_U_* = *m_V_*. Further, following Youssefpour et al. (2012), we assume the apoptosis and mitosis rates of the TCs are equal: *a_V_* = *m_V_*. These assumptions imply that the TC populations dies out if *k*(0) ≤ 1, which is equation (6) for this case. When combined with our earlier assumptions regarding *k*(*P*) and with our chosen form for *k*(*P*) [equation (3)], we see that in our model TCs are always doomed to die out in the absence of CSCs. Our assumptions regarding the mitosis rates and TC apoptosis rate also simplify the form of the slow manifold to

(10) $$M:=\left\{\left(U,V\right):\left(1-k\left(P\right)\right)V=k\left(P\right)U,P=U+V\right\}$$

for all simulations.

Whenever we apply radiation therapy, we assume no difference in the ability of CSCs and TCs to withstand lethal single track damage. Thus, we use the same *α* value for both cell types. As mentioned previously, we set *β* = 0 for CSCs to simulate perfect repair of two-track non-lethal damage.

All numerical simulations are carried out in Maple™, using the dsolve ODE solver employing the rfk45 numerical method. For every simulation, the initial conditions are (*U*_0_, *V*_0_) = (0.1, 0.1), and therapy begins on the two-hundredth day. These settings allow the tumor system to hit the slow manifold, *M*, before treatment begins, in each of our simulations.

To prevent negative volume fractions during numerical simulation, we introduce a simple cutoff function

(11) $$G\left(x\right)=\left\{\begin{array}{cc} 1,\; & x>\lambda \\ 0,\; & x\leq \lambda \end{array}\right.$$

where *λ* is chosen to allow the TCP to approach one before the cutoff is imposed. The system we use for numerical simulation, incorporating the cutoff function is

$$\begin{array}{llll} \overset{{}^{\circ}}{U}\left(t\right) & =\left(2p-1\right)mk\left(P\left(t\right)\right)U\left(t\right)G\left(U\left(t\right)\right) & & \\ \overset{{}^{\circ}}{V}\left(t\right) & =2\left(1-p\right)mk\left(P\left(t\right)\right)U\left(t\right)G\left(U\left(t\right)\right) & & \\ & \;+mk\left(P\left(t\right)\right)V\left(t\right)G\left(V\left(t\right)\right)-mV\left(t\right)G\left(V\left(t\right)\right) \end{array}$$

where *m* = *m_U_* = *m_V_* = *a_V_*.

As a measure of treatment success, we calculate the TCP [equation (9)] using the value of *U* obtained at the end of treatment, which is defined as the latter of: (a) the completion of the final radiation fraction, and (b) the point in time when *p*(*t*) reaches 0.5, after differentiation therapy has ended. This second point (b), accounts for the effect of lingering differentiation promoter, after the promoter is no longer being applied.

## 3 Results

We summarize the chosen parameter values in Table 1 and we give relevant references and explanations in the following subsections.

**Table 1:** **Summary of model parameters for the three cancers studied here**.

Cancer	*α/β* [Gy]	*α* [Gy^−1^]	*β* [Gy^−2^]	*m* [day^−1^]	R [cm]	d [Gy]	Max D [Gy]
Head and neck	10	0.35	0.035	ln 2/3	1.5	2.53	63.25
Brain cancer	12	0.3	0.025	ln 2/3.9	1.9	3.8	57.5
Breast	2.88	0.08	0.0027	ln 2*/*8.2	0.25	2.26	65.54

*All radiation treatment schedules are standard treatments, with one fraction each week day and weekends off. Further references and explanations are given in the text below*.

### 3.1 Head and neck cancer tumor simulations

**Figure 2:**
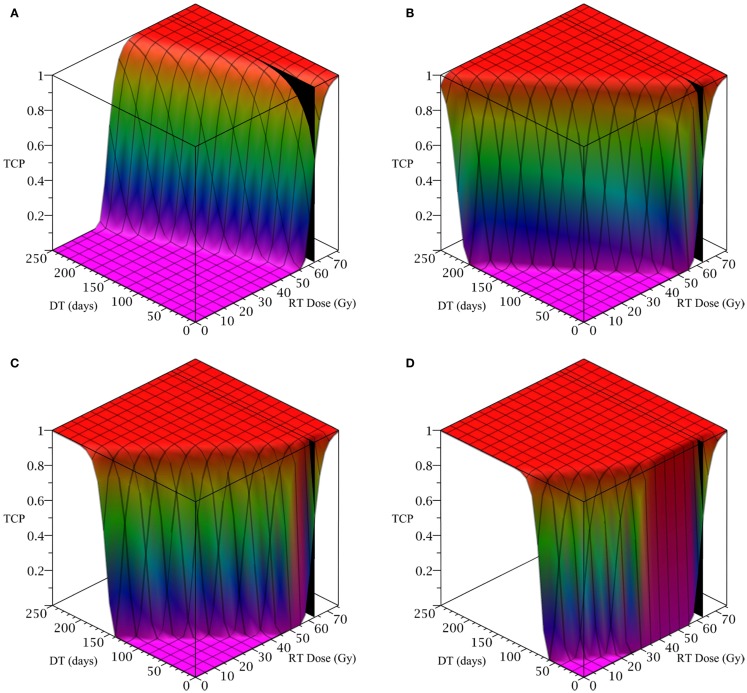
**(Head and neck) TCP for various regimens of differentiation therapy (DT), radiation therapy (RT), and combination therapy, as applied to a simulated head and neck cancer tumor**. The RT protocol and tumor parameters are described in Table 1. **(A)** Simulations with CSC sensitivity to DT, *ψ* = 0.5. Thus, the probability of CSC self-renewal, *p* > 0.40. **(B)** Simulations with *ψ* = 2. Thus, *p* > 0.30. **(C)** Simulations with *ψ* = 5. Thus, *p* > 0.25. **(D)** Simulations with *ψ* = 50. Thus, *p* > 0.205.

To simulate ahead and neck tumor, we choose the following parameter values for the LQ model: an *α/β* ratio of 10 Gy and an *α* value of 0.35 Gy^−1^ (Fowler, 2010). We set the mitosis rates of the TCs and CSCs to ln 2*/*3 day^−1^, using a cell doubling time of 3 days as per Fowler (2010). We note that this is an estimate of cell doubling time for cells undergoing cytotoxic treatment, which tend to have shorter doubling times than untreated cells (Fowler, 2010). For the radius of the domain of interest, *R*, we choose 1.5 cm. All simulations of radiation therapy use fraction sizes of 2.53 Gy, delivered once per day, on weekdays only. We used one of the optimized head and neck radiation schedules recommended by Fowler as a constraint on radiotherapy, which is 25 fractions of 2.53 Gy each, for a total of 63.25 Gy delivered over 32 days (Fowler, 2010). This schedule is optimized to satisfy a late tissue constraint of 70 Gy $\text{EQ}{\text{D}}_{3/2}$ and an acute mucosal constraint of 51 Gy $\text{EQ}{\text{D}}_{10/2}$ while delivering the maximum possible BED to the tumor, given the chosen fraction size and weekday only schedule (Fowler, 2010). We indicate the position of this schedule in Figure 2 using a black plane at 63.25 Gy, which we take as our constraint on radiation therapy.

The only parameter not yet specified is the sensitivity *ψ* toward the differentiation promoter and we have no experimental data available. In Figure 2, we show four simulations of the tumor control probability for four different sensitivities *ψ* = 0.5, 2, 5, 50 which covers a wide range of possible values. The *x*-axis denotes the duration of the differentiation treatment and the *y*-axis denotes the total radiation dose. The black plane indicates the maximum tolerable radiation dose in this particular treatment. The colored plane is the tumor control probability (TCP). We see in all four Figures that the TCP is 0 near the origin and it rises sharply to values close to one as both treatment modalities are increased. In Figure 2B for example, we see that radiation alone reaches a TCP of about 60% for the maximum dose. In combination with differentiation therapy of 50 days, we observe treatment success already at total dose of 40 Gray. This effect is more pronounced for higher sensitivity parameter *ψ*. Notice that the curve for 0 DT days is the same in all four figures.

A good quantitative measurement for efficiency of a treatment is the TCP = 50% value. To illustrate how the treatment regimens change for a fixed TCP, we list a few treatment regimens that result in a 50% TCP in Table 2. We see that for large enough sensitivity *ψ*, the total radiation dose can be drastically reduced if differentiation therapy is applied.

**Table 2:** **A selection of head and neck cancer tumor treatment parameters resulting in TCP ≈ 0.5**.

DT sensitivity, *ψ*	DT duration (days)	Total radiation (Gy)	TCP
N/A	0	63.25	0.581
0.5	9	60.72	0.498
	29	58.19	0.506
2	18	53.13	0.492
	35	48.07	0.490
5	20	45.54	0.508
	39	37.95	0.486
50	4	35.42	0.498
	36	15.18	0.505

*These are selected from simulations using the model parameters in Table 1 where differentiation therapy is varied in increments of a single day, from 0 to 60 days, and radiation therapy in increments of a single fraction, from 0 to 75.9 Gy total radiation. The TCP without differentiation therapy is given as a point of reference*.

### 3.2 Brain cancer simulations

**Figure 3:**
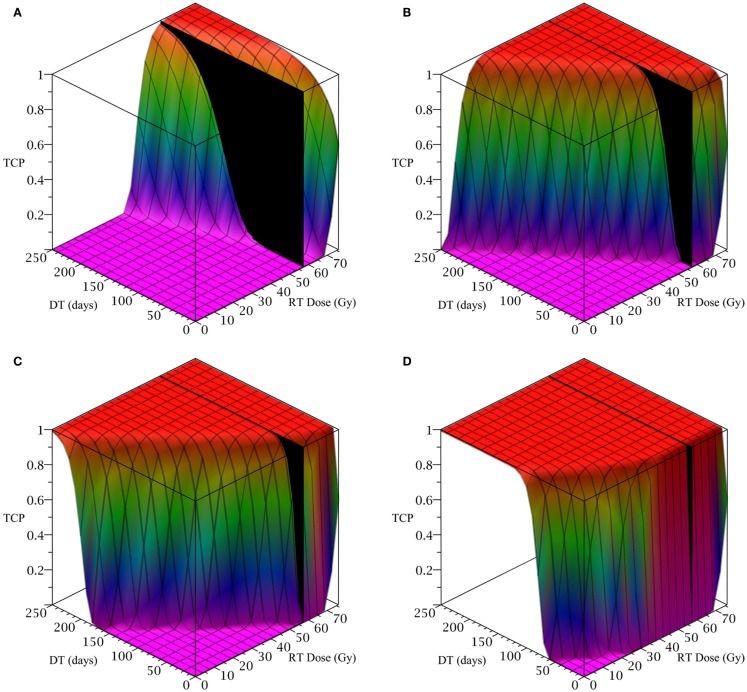
**(Brain cancer) TCP for various regimens of differentiation therapy (DT), radiation therapy (RT), and combination therapy, as applied to a simulated brain cancer with average parameter values**. The RT protocol and tumor parameters are described in Table 1. **(A)** Simulations with CSC sensitivity to DT, *ψ* = 0.5. Thus, the probability of CSC self-renewal, *p* > 0.40. **(B)** Simulations with *ψ* = 2. Thus, *p* > 0.30. **(C)** Simulations with *ψ* = 5. Thus, *p* > 0.25. **(D)** Simulations with *ψ* = 50. Thus, *p* > 0.205.

To simulate a brain cancer, we use an average *α/β* ratio of 12 Gy and *α* value of 0.3 Gy^−1^, as estimated by Yuan et al. (2008) for brain cancers (primary tumors as well as brain metastatic cancers). This gives a *β* value of 0.025 Gy^−2^. For the radius of the domain of interest, *R*, we use 1.9 cm, which is roughly the radius of a sphere of volume 28.8 cm^3^, the volume of a brain metastatic cancer arising from non-small-cell lung cancer, reported in the same paper (Yuan et al., 2008). For the CSC and TC growth rates, we use ln 2*/*3.9 day^−1^, where 3.9 is an estimate of the mean potential doubling time of brain metastatic cancer originating from various primary cancers, as measured by flow cytometry (Struikmans et al., 1997). All simulations involving radiation use a fraction size of 3.8 Gy, delivered once per day on weekdays only. This fraction size is listed by Yuan et al. (2008) as part of a hypofractionated stereotactic radiotherapy regimen involving 15 fractions, and it approaches the radiation tolerance for normal brain tissue. We take the total dose of 57.5 Gy listed by Yuan et al. (2008) as our constraint on radiation therapy, which appears as a black plane in Figure 3.

The results as documented in Figure 3 are very similar to those for the head and neck cancer. One difference is that without any differentiation therapy, the cancer cannot be controlled by radiation alone. At least not within the given parameter values. In Table 3 we list some TCP 50% values for this case.

**Table 3:** **A selection of brain cancer treatment parameters resulting in TCP ≈ 0.5**.

DT sensitivity, *ψ*	DT duration (days)	Total radiation (Gy)	TCP
N/A	0	76.0*	0.602
0.5	17	72.2*	0.501
	50	68.4*	0.504
2	19	64.6*	0.504
	46	57.0	0.502
5	17	57.0	0.518
	35	49.4	0.500
50	3	41.8	0.500
	47	11.4	0.503

*These are selected from simulations using the model parameters in Table 1 where differentiation therapy is varied in increments of a single day, from 0 to 60 days, and radiation therapy in increments of a single fraction, from 0 to 76 Gy total radiation. The TCP without differentiation therapy is given as a point of reference*.

*Violates radiation constraint of 57.5 Gy.

### 3.3 Breast cancer tumor simulations

**Figure 4:**
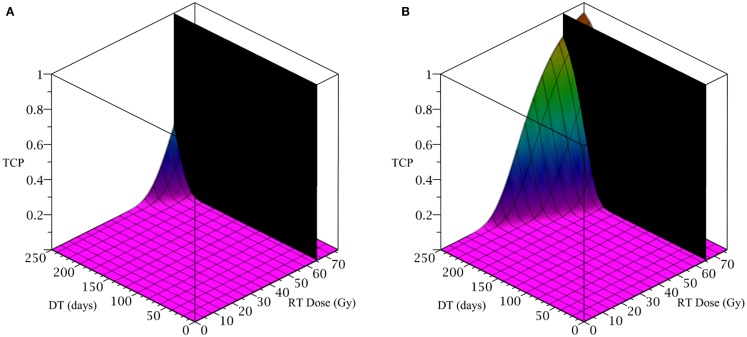
**(Breast) TCP for various regimens of differentiation therapy (DT), radiation therapy (RT), and combination therapy, as applied to a simulated breast cancer tumor**. The RT protocol and tumor parameters are described in the corresponding result section. **(A)** Simulations with CSC sensitivity to DT, *ψ* = 5. Thus, the probability of CSC self-renewal, *p* > 0.25. **(B)** Simulations with *ψ* = 50. Thus, *p* > 0.205. We do not include graphs for simulations with *ψ* = 0.5 or *ψ* = 2, as they result in TCP ≈ 0 for all treatment regimens tested.

To simulate the treatment of a small breast tumor, perhaps remaining after the resection of a large tumor, we choose *R* = 0.25 cm. The CSC and TC growth rates are set to ln 2*/*8.2 day^−1^, where 8.2 is the median potential doubling time of human breast tumors measured by Rew et al. (1992) using flow cytometry. Plausible parameter values for the LQ model [equation (8)] are taken from Qi et al. (2011): *α/β* = 2.88 Gy and *α* = 0.08 Gy^−1^. We use a fraction size of 2.26 Gy, delivered once per day on weekdays only. Our radiation constraint, indicated by a black plane in Figure 4, is 65.54 Gy, which corresponds to 29 fractions, the maximum number of fractions that satisfy the late tissue constraint of 70 Gy $\text{EQ}{\text{D}}_{3/2}$ and the acute mucosal constraint of 51 Gy $\text{EQ}{\text{D}}_{10/2}$ given in Fowler (2010).

Since the breast tumor in this example is late responding (low *α/β*-ratio), it is very difficult to control the cancer with radiation alone. The maximum tolerable dose of 65.54 Gy is reached much earlier than the TCP shows any growth. Using radiation in combination with differentiation therapy gives some hope that the cancer can be eradicated. Provided, however, that the CSC cells are sensitive enough to the differentiation promoter. In Table 4 we list a few TCP 50% values.

**Table 4:** **A selection of breast cancer tumor treatment parameters resulting in TCP ≈ 0.5**.

DT sensitivity, *ψ*	DT duration (days)	Total radiation (Gy)	TCP
5	238	76.84*	0.499
	247	72.32*	0.505
50	204	74.58*	0.503
	222	63.28	0.500

*These are selected from simulations using the model parameters in Table 1 where differentiation therapy is varied in increments of a single day, from 180 to 250 days, and radiation therapy in increments of a single fraction, from 0 to 76.84 Gy total radiation. We use a minimum of 180 days of differentiation therapy, as the TCP remains near 0 until this level of DT is applied (see Figure 4)*.

*Violates radiation constraint of 65.54 Gy.

## 4 Discussion

Current treatment modalities of cancer include surgery, radiation, chemotherapy, immuno-therapies, hormone therapies, and differentiation therapies. All of these methods have distinct advantages and limitations and clinicians often combine various methods to obtain the best results. In fact, in most cases a surgical removal or a radiation treatment is followed by chemotherapy. However, if chemotherapy is based on a single cytotoxic agent then the sensitive part of the tumor is killed but the resistant cell population persists; leading to chemo-resistance (Swierniak et al., 2009). The sensitivity to ionizing radiation can also vary in a tumor, where quiescent cells, or stem cells are less radiosensitive than cells that are actively proliferating (Kim and Tannock, 2005; Pajonk et al., 2010). Differentiation therapy describes the attempt to force stem cells into differentiation to increase their sensitivity to treatment agents (Leszczyniecka et al., 2001; Sell, 2004). This idea is conceptually intriguing and it is our attempt in this paper to quantify the possible benefit for three specific cases: head and neck cancer, brain cancers, and breast cancer.

Our results are based on a mathematical model for the dynamics of cancer stem cells (CSC) and non-stem cancer cells (TC). The model is derived from previous models of Youssefpour et al. (2012) and Hillen et al. (2013) and it includes control through differentiation therapy and radiation treatment. The benefit of a given treatment is computed using the (Poissonian) tumor control probability (TCP).

We found very good references to most of the model parameters such as growth rates, doubling times, tumor volumes, and radiation sensitivities (see Table 1). However, we were not able to find good measurements for the sensitivity parameter *ψ*. Differentiation therapy alone has been used successfully in several cases. For example, about 70% of acute promyelocytic leukemia can be controlled by ATRA-therapy (all-trans-retinoic acid, Sell, 2004). Melanoma can be treated with the differentiation promoter cocktail of inferon-β and mezerein (Leszczyniecka et al., 2001); and Lander et al. (2009) and Youssefpour et al. (2012) suggest the use of the differentiation promoter TGF-*β*. However, to our knowledge, the effect of these promoters has never been quantified. Hence we reside to explore a wide range of possible sensitivities *ψ*.

In each case we found a clear advantage of combination therapy, where differentiation therapy drastically reduces the total radiation dose. We are able to confirm the findings of Youssefpour et al. (2012) for the cases of head and neck cancer, brain cancers, and breast cancer data. For future studies it is important to get estimates for the sensitivity *ψ* and we hope that research groups around the world might be able to identify this in the future.

It should be noted that the above model is over-simplistic to fully model a growing tumor. For the brain-tumor, for example, the spatial extent of the tumor is a dominating problem for treatment. The knowledge of an optimal combination therapy schedule is only useful if the overall treatment volume is known. It is the focus of ongoing research to identify a suitable treatment volume (see Konukoglu et al., 2010; Painter and Hillen, 2013). In addition, the immune response will be an important player in each of the tumors mentioned above. As discussed by Hanahan and Weinberg (2011), the immune system can be both, tumor promoting and tumor inhibiting and the complex interactions are not fully understood. When we face all these additional difficulties, it appears as an advantage to have a simple sub-model, such as (4, 5), which clearly and consistently shows the benefit of combination therapy for a wide range of parameters and a selection of different tumors. This suggests that a combination of differentiation therapy and radiation therapy should be considered as a serious alternative.

## Conflict of Interest Statement

The authors declare that the research was conducted in the absence of any commercial or financial relationships that could be construed as a potential conflict of interest.
